# Detection of Enteric Viruses on Strawberries and Raspberries Using Capture by Apolipoprotein H

**DOI:** 10.3390/foods10123139

**Published:** 2021-12-18

**Authors:** Anthony Lévesque, Eric Jubinville, Fabienne Hamon, Julie Jean

**Affiliations:** 1 Department of Food Sciences, Institute of Nutrition and Functional Foods (INAF), Université Laval, Quebec, QC G1V 0A6, Canada; anthony.levesque.2@ulaval.ca (A.L.); eric.jubinville.1@ulaval.ca (E.J.); 2 Food Molecular Biology RD Department, bioMérieux, 38000 Grenoble, France; fabienne.hamon@biomerieux.com

**Keywords:** capture, detection, hepatitis A virus, human norovirus, apolipoprotein H, beta-2-glycoprotein-1, magnetic beads

## Abstract

Human noroviruses (HuNoVs) and the hepatitis A virus (HAV) are the main viral causes of foodborne illness worldwide. These viruses are frequently transmitted via fresh and frozen berries, such as strawberries and raspberries. ISO 15216:1 (2017), currently the preferred method for their detection, involves several steps and is time-consuming. Apolipoprotein H (ApoH) has been shown to have a strong affinity for several microorganisms, including HuNoVs. In this article, we report an ApoH-based method of capturing the HAV and HuNoVs adherent to berries and concentrating them for assay. The limit of detection of both viruses suspended in a buffer was low. On strawberries, the HAV was detected down to 10^4^ genome copies/25 g in 100% of cases and down to 10^3^ genome copies/25 g on raspberries in 50% of cases. This sensitivity was not significantly different from that of the ISO method 15216:1 (2017). HuNoV GII.4 was more difficult to detect using the ApoH method. The ApoH CaptoVIR kit does, nevertheless, appear to be usable in the near future as a single-test, multiple-detection method for viruses on fresh and frozen berries.

## 1. Introduction

Human noroviruses (HuNoVs) and hepatitis A virus (HAV) are the main viral causes of diarrheal and non-diarrheal enteric illness [1]. In 2010, 120 million cases of HuNoVs and 14 million cases of HAV were reported worldwide [1]. A concerning increase in the percentage of cases involving foodborne transmission has occurred in recent years [2,3]. Soft fruits and vegetables are frequent vehicles of transmission [4] and have been implicated in several outbreaks around the world [2]. Between 1983 and 2018, 68 outbreaks of HAV or HuNoVs and over 18,000 illnesses were associated with berries [2]. Frozen berries, especially strawberries for HAV and raspberries for HuNoVs, were the main vectors of these outbreaks [2]. Complete elimination of these viruses on berries would be a welcome development, but this is difficult to achieve with current inactivation methods. In the meantime, prevention of outbreaks relies on viral detection and product recall. Since viruses are usually present in very low numbers on berries, concentrating them prior to detection is a critical step. In addition, berries release multiple inhibitors of the real-time polymerase chain reaction (qPCR) [5], and these need to be removed to avoid false negative assay results. The current gold standard method for detecting viruses on berries is the ISO method 15216:1 (2017), which involves a concentration step based on precipitation with polyethylene glycol/sodium chloride (PEG/NaCl) [6]. This method can be performed routinely but requires several hours and certain technical skills. Other methods of concentration based on affinity, such as capture with ligands [7] or antibodies [8,9], or on physical separations, such as ultrafiltration [8,9] and ultracentrifugation [10], are currently in development. However, physical methods often perform poorly when the food matrix is complex (inclined to clog ultrafiltration membrane pores) [11] and few methods concentrate both HuNoVs and HAV with high specificity.

Apolipoprotein H (ApoH, also known as beta-2-glycoprotein I) is a plasma protein involved in antiphospholipid syndrome (APS syndrome) [12]. This protein has a broad-spectrum affinity, including for hepatitis B and C viruses [13,14], HuNoVs, and many Gram-positive and Gram-negative bacteria [15]. To the best of our knowledge, its affinity for HAV is unknown [15], and the possibility of using it in magnetic bead form for concentrating and detecting the HAV and HuNoVs on berries in a single method should therefore be investigated.

The purpose of this study was to investigate the affinity of ApoH for HAV and to determine the limit of detection of HAV and HuNoV GII.4 on fresh and frozen strawberries and raspberries using ApoH as a capture and concentration step. The ApoH method was compared to the ISO method 15216:1 (2017) using fresh strawberries and raspberries.

## 2. Materials and Methods

### 2.1. Cell Culture and Virus Production

The cytopathogenic HAV strain HM-175 was cultured on renal rhesus macaque FRhK-4 cells [16,17]. The cells were grown as described previously [18] on Dulbecco modified Eagle’s medium (DMEM) with 10% (*v*/*v*) fetal bovine serum, 200 mM l-glutamine, essential amino acids, 1 M *N*-2-hydroxyethylpiperazine-*N*′-2-ethanesulfonic acid (HEPES), and penicillin–streptomycin (all culture medium products from Wisent Inc., Saint-Jean-Batiste, QC, Canada) in cell-adherent culture flasks (75 cm^2^). They were infected with HAV at a multiplicity of infection (MOI) of 0.01 and incubated at 37 °C and 5% CO_2_ for 90 min. The medium was changed to 2% fetal bovine serum (maintenance medium) for incubation until 70–80% cell confluence was reached (~4–5 days). The cells were then lysed by subjecting them to 3 freeze/thaw cycles (−80 °C then 37 °C in an incubator). The supernatant was centrifuged at 1000× *g* for 10 min and aliquoted [16,17]. Viral titer was measured using reverse-transcriptase qPCR(RT-qPCR).

HuNoV was provided by the Centre Hospitalier Universitaire Dr-Georges-L.-Dumont Laboratoire de Microbiologie (Moncton, NB, Canada) in suspensions obtained by centrifuging fecal samples diluted 1:10 in phosphate-buffered saline (1× pH 7.0) at 3000× *g* for 30 min at 4 °C. The suspensions were aliquoted upon receipt, sent for sequencing, and stored at −80 °C.

### 2.2. Sequencing of HuNoVs

The norovirus was sequenced at the Plateforme d’Analyses Génomiques of the Institut de Biologie Intégrative et des Systèmes (PAG-IBIS, Université Laval, Québec, QC, Canada) using the BigDye™ Terminator v3.1 cycle sequencing kit (Applied Biosystems, Foster City, CA, USA), an ABI 3500 series analyzer (capillaries 50 cm), and Chromas v2.6.6; and it was aligned using BioEdit v7.0.5.3 and BLAST with the Norovirus Genotyping Tool v2.0 (https://www.rivm.nl/mpf/typingtool/norovirus/ (accessed on 16 December 2021)). The sequence of NoV GII.4 used in this study can be retrieved using the GenBank number MZ357344.

### 2.3. Viral Capture Using ApoH

#### Virus Test Suspensions

A suspension of HAV and/or HuNoV containing about 10^4^ genome copies/µL was added to distilled water to obtain the experimental titers of 10^5^ genome copies in 1 mL or 40 mL of sample. These dilutions were used within 30 min.

### 2.4. Artificial Contamination of Fruits

About 10^5^ viral genome copies suspended in 100 µL were added to 25 g of fresh or frozen strawberries or raspberries in a sterile stomacher bag. All experiments were performed in triplicate. The sample was kept at 4 °C for 30 min before the elution of the virus.

### 2.5. Elution of Virus from Fruit

CV-1 buffer (40 mL; ApoH CaptoVIR kit (Catalog number #MP10022-100T, ApoH Technologies, La Grande Motte, France)) was added to the stomacher bag, which was then shaken gently for 10 min at room temperature on a homemade device at about 30 cycles per minute. The free liquid was recovered in a 50 mL tube and kept at 4 °C for 30 min until viral capture.

### 2.6. Viral Capture

ApoH-coated beads in 10 µL of standard suspension (ApoH Technologies) were added to the CV-1 buffer, which was then shaken on a rotary mixer at 45 rpm for 15 min at 4 °C. The beads were trapped by placing a neodymium magnet on the outside of the tube. The free liquid in the tube was discarded, the magnet was pulled off, and the beads were resuspended in 2 mL of NucliSENS^®^ lysis buffer (bioMérieux, Marcy-l’Étoile, France).

### 2.7. Viral RNA Extraction

The suspension of ApoH magnetic beads in lysis buffer was vortexed, held at 56 °C for 30 min, and then centrifuged at 1800× *g* for 2 min. Viral RNA was then extracted according to the procedure recommended by the manufacturer of the NucliSENS^®^ miniMAG^®^ system (bioMérieux), eluted in 100 µL of NucliSENS^®^ elution buffer 3, stirred at 1400 rpm for 5 min at 60 °C in a thermomixer (Eppendorf, Hamburg, Germany), and then held for 1 min on a magnetic stand (bioMérieux). The supernatant was stored at −80 °C until RT-qPCR analysis.

### 2.8. RT-qPCR

Viral RNA was quantified using the iTaqTM Universal Probe One-Step RT-qPCR kit (BioRad, Hercules, CA, USA). The primers (Applied Biosystems), probes, and standard plasmid RNA (IDT, Coralville, IA, USA) are listed in ISO protocol 15216:1 (2017) [6]. An Applied Biosystems 7500 Real-Time PCR Thermal Cycler was used to produce the following temperature profile: 10 min at 50 °C, 3 min at 95 °C, 45 cycles of 15 s at 95 °C, and 30 s at 60 °C. Standard curves were generated using 10-fold serial dilutions of standard plasmid RNA produced for both viruses. The R^2^ value of each standard curve was higher than 0.98 and the slopes were between −3.10 and −3.60. ROX was used as the reference dye. An automatic threshold was used to determine cycle quantification (Cq) and ∆Cq values. The number N of viral genome copies in the sample was calculated as follows:

N = 100 × 10R

where R is ∆Cq/M and M is the slope of the standard curve.

### 2.9. Limit of Detection

The limit of detection of HAV was determined in 1 mL and 40 mL of CV-1 buffer (ApoH Technologies) and on the four food matrices, whereas the limit of detection of the NoV was determined only on the foods. Samples were loaded with 10^5^, 10^4^, 10^3^ or 10^2^ genome copies. Positive and negative extraction controls were run for each experiment (a positive control at each concentration). Results were scored as detected or undetected. A sample was considered positive when the Cq was ≤40.

### 2.10. Comparison of the ISO and ApoH Methods

The capture efficiencies of the ISO 15216:1 (2017) and ApoH methods were compared for fresh fruit only (performed in triplicate). For this purpose, HAV and NoV GII.4 were loaded simultaneously at the lowest concentration determined in the limit of detection experiments. The ISO method was performed as described in the standard protocol [6] with mengovirus as the procedural control. Positive in triplicate and negative controls were run at the concentration used. Results are expressed as the log percent genome copies recovered (percent recovery).

### 2.11. Statistical Analysis

All experiments were performed independently in quadruplicate. The ISO and ApoH were compared in terms of genome copies measured by RT-qPCR. The percent recovery of the virus was based on the ratio of the sample to its positive control, as per the ISO method [6]. Significant differences were based on two-tailed, unpaired *t*-tests at 95% confidence.

## 3. Results

### 3.1. Investigation of the Affinity between Hepatitis A Virus and Apolipoprotein H

Suspended HAV bound readily to ApoH-coated beads, and the volume of CV-1 buffer had no impact on the recovery and detection of the virus (Table 1). The HAV genome was detected over the entire range of copy numbers, from 10^5^ to 10^2^ per sample. However, the number of positive samples was higher at 10^5^ and 10^4^ (100%) than at 10^3^ (75%) or 10^2^ (25%). No sample containing only 10 genome copies was found positive (data not shown).

### 3.2. The Limit of Detection of Hepatitis A Virus on Berries

HAV, presumed to be adherent to the fresh and frozen strawberries and raspberries, was captured on ApoH beads (Table 2). There was no significant difference between the numbers detected on fresh and frozen strawberries, reaching 100% in both cases at 10^5^ and 10^4^ genome copies per sample. However, freezing did make a difference in the case of raspberries, making the adherent HAV practically undetectable. The HAV on strawberries at 10 genome copies per sample was not detectable (data not shown).

### 3.3. The Limit of Detection of Norovirus GII.4 on Berries

NoV GII.4 on fresh and frozen strawberries and raspberries was captured on ApoH beads (Table 3). Freezing the strawberries did not interfere with the capture, certainly not at 10^5^ genome copies per sample. However, NoV GII.4 was almost undetectable on frozen raspberries and was detected on fresh raspberries only when the genome copy number loaded was 10^5^ or 10^4^.

### 3.4. Comparison of the ISO and ApoH Methods Using Strawberries

**Figure 1:**
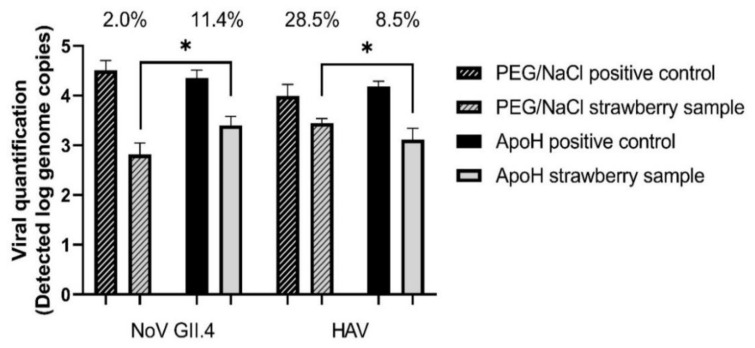
Simultaneous detection of HAV and NoV GII.4 on fresh strawberries using PEG/NaCl or ApoH beads to concentrate the viruses. Initial viral load at 1 × 10^4^ genome copies/sample for HAV and at 1 × 10^5^ genome copies/sample for NoV GII.4. Asterisk indicates statistically significant differences (*p* ≤ 0.05) between viral recuperation of ApoH method and ISO method based on the unpaired, two tailed *t*-test.

Based on the results in Table 2 and Table 3, fresh berry samples were loaded with 10^4^ HAV genome copies and 10^5^ NoV GII.4 genome copies. Using the ISO PEG/NaCl method, we recovered 2% of the NoV and 28.5% of the HAV (Figure 1). Recovery of the mengovirus from all the samples was greater than 1%, which is the limit of validity of the ISO method. Using the ApoH method, we recovered 11.4% of the NoV and 8.5% of the HAV. The difference between these two methods of viral extraction was significant for both viruses.

### 3.5. Comparison of the ISO and ApoH Methods Using Raspberries

**Figure 2:**
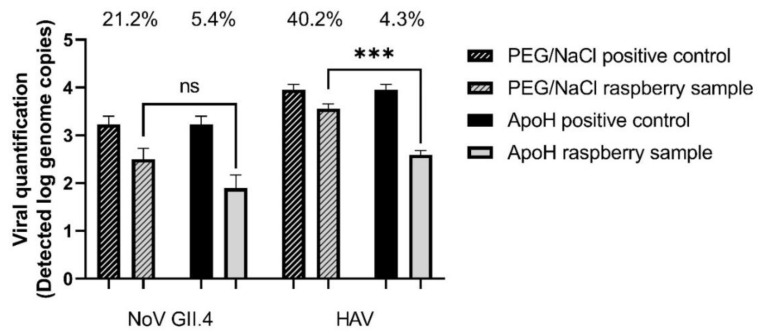
Simultaneous detection of HAV and NoV GII.4 on fresh raspberries using PEG/NaCl or ApoH beads to concentrate the viruses. Initial viral load at 1 × 10^5^ genome copies/sample and NoV GII.4. Three asterisks indicate statistically significant differences (*p* ≤ 0.001), and ns indicates no statistically significant differences between the viral recuperation of the ApoH method and the ISO method based on the unpaired, two tailed *t*-test. Statistical significance is based on the unpaired, two-tailed *t*-test.

Fresh raspberries were loaded with 10^4^ HAV and 10^4^ NoV GII.4 genome copies (based on Table 2 and Table 3). Using the ISO PEG/NaCl method, we recovered 21.2% of the NoV and 40.2% of the HAV genome copies (Figure 2). Recovery of the mengovirus was greater than 1% in all samples. Using the ApoH method, we recovered 5.4% of the NoV and 4.3% of the HAV genome copies. The difference between the two methods was not significant for NoV but was for HAV (*p* ≤ 0.001).

## 4. Discussion

It is important to develop a universal method to concentrate foodborne viruses and overcome the limitations inherent in the ISO method 15216:1 (2017). We proposed the use of the broad-spectrum affinity protein ApoH, which is composed of five closely related domains, of which the fifth is rich in lysine residues [19], producing a strong positive charge at one end of the molecule, the active form of which is hook-shaped [20]. The exact role that this peculiar shape plays in the affinity of ApoH for viruses remains undetermined. The affinity of ApoH for anionic phospholipids is known to contribute to its involvement in APS disease [21] and could explain its binding of enveloped viruses such as HBV [13], HCV [14], and the human immunodeficiency virus [22]: all known to have membranes containing phosphatidylserine [23]. However, ApoH also binds microorganisms and naked viruses by a mechanism that remains unknown. We hypothesized that ionic interactions must be involved. The isoelectric points of the NoV GII and the HAV capsids fall, respectively, in the ranges 5.5–6.9 [24] and 2.8–5.5 [25,26]. At a neutral pH (in CV-1 buffer), these viruses should bear a negative charge and, therefore, bind readily to ApoH. One reason poor results were obtained with frozen raspberries could be that the virus was eluted at a pH that dropped as low as 3.0, compared to 5.0 for the fresh fruit. This drop in pH was smaller for the strawberry samples: 6.0 for fresh and 4.0 for frozen. Viral particles can have a neutral charge at a pH of 5 or 6 but would certainly have a positive charge at a pH of 3.0 or 4.0 (maybe neutral in the case of HAV). Shifts in surface charge, therefore, could explain the differences in the results obtained with strawberries and raspberries. It was shown that at a pH of 4.0, HuNoV particles tend to aggregate [27], which could make capture by ApoH more difficult. If the problem were this simple, adjusting the pH to neutrality before adding the ApoH beads to the suspension of the virus eluted from fruits should improve the results. Another important factor may be the detersive power of the CV-1 buffer compared to the TAS buffer used with the ApoH CaptoVIR kit (Catalog number #MP10022-100T, ApoH Technologies, La Grande Motte). The composition of this commercial buffer is a trade secret. The CV-1 buffer appeared to dislodge the viruses more effectively from the berries (data not shown). Additional studies are required to confirm these explanations.

It is important to note the difference between our contamination protocol of frozen fruits compared to the natural contamination of berries. Berries will be contaminated prior to the freezing steps in food industries when they are fresh. So, to be as realistic as possible, fresh fruit should be contaminated and then frozen. On the other hand, if performed under laboratory conditions, freezing should be performed using the food industry’s freezing protocol. The equipment used for this process is not easily accessible and cannot be used for experimental contaminations with foodborne pathogens. Moreover, under laboratory conditions, after the artificial contamination of fresh berries, the freezing process is slower than the one used in the food industry. This protocol could damage the matrix and would release even more inhibitors, adding fastidious purification steps.

Based on our comparison between the ISO method 15,216:1 (2017) and the ApoH method, the recovery of the HuNoV with ApoH seems to be more efficient on strawberries (Figure 1), while the data on raspberries are similar (Figure 2). However, the ISO method was better at recovering the HAV from both berries. This could be due to using TGBE buffer for viral elution in the ISO method, the pH thus starting at 9.5 and not decreasing much whether testing strawberries or raspberries. In addition, during the concentration step using PEG/NaCl, the pH is brought back to 7.0, which may be ideal, depending on the isoelectric point of the viruses. This suggests that adjusting the pH could improve the viral capture by the ApoH method. Furthermore, at pH 5.0, the pectin present in berries, particularly in raspberries, forms a strong gel [28]. Unlike the ISO method, which includes the addition of pectinase as well as adjusting the pH to 7.0 and is unaffected by the presence of pectin, the ApoH method could be affected by pectin. On the other hand, magnetic separation supposedly removes the virus regardless of the presence of inhibitors. In addition, we were able to capture more NoV GII.4 from the strawberries using the ApoH method, which suggests that inhibitors other than pectin affect the quantification step, giving a slight advantage to ApoH. It is also important to consider the potential presence of other microorganisms in the HuNoV stocks, such as Gram-negative bacteria, which could compete for ApoH affinity sites and decrease HuNoV recovery [15]. Different inhibitors could also come from the diluted fecal matter in the HuNoV stock solution. This is different from the HAV stock solution that was produced from a cell culture protocol. Although not large, the difference is significant. The use of the ApoH CaptoVIR kit was described by only one published study, in which only 1 mL of suspension was processed and with only one virus (NoV GI or GII or Tulane virus) per test [15]. Our recoveries of NoV GII from a virus mixture were slightly lower.

The ApoH method is simpler and faster than the ISO 15216:1 (2017) method. Viral elution and capture take approximately 50 min compared to the ISO method’s 3 h or more. This alone would make the ApoH method more suitable for routine use. It requires no chloroform/butanol or any noxious chemical and does not rely on centrifuging. Although it currently costs more than the ISO method, even at 15 $/sample, the ApoH method is advantageous due to the time saved and the ease of handling. Our results suggest that the ApoH method will facilitate and simplify the analytical workflow compared to the ISO method.

In conclusion, we demonstrated that ApoH CaptoVIR kit could be used to detect foodborne viruses in berries. Its affinity with the HAV and HuNoV make this new tool an interesting candidate in the world of foodborne viral detection methods. Furthermore, the methodology used by this kit is faster and shows a similar viral recovery rate compared to the ISO method. To the best of our knowledge, this is the first study reporting such an affinity with the HAV. Additionally, our data suggest that the ApoH CaptoVIR kit has the potential to be a single-test, multiple-detection method for viruses in fresh and frozen berries. However, further studies are necessary to optimize the method and, thus, detect viruses efficiently at lower concentrations. Investigating its potential on other food matrices, including bivalve mollusks, ready-to-eat foods, and other berries is also required. Overall, our study demonstrated a proof of concept of the potential of ApoH viral capture on food matrices.

## Figures and Tables

**Table 1:** Detection of the HAV using capture by ApoH beads in CV-1 buffer.

Sample Volume	Genome Copies/Sample			
	10^5^	10^4^	10^3^	10^2^
	Number of Positive Samples/Number of Samples Tested			
1 mL	4/4	4/4	3/4	1/4
40 mL	4/4	4/4	3/4	1/4

Samples are considered positive if the Cq is less than or equal to 40. For each concentration and volume, four samples were treated with the ApoH methods. No statistical analysis was performed.

**Table 2:** Detection of HAV on berries using capture by ApoH beads.

Food	Genome Copies/Sample			
	10^5^	10^4^	10^3^	10^2^
	Number of Positive Samples/Number of Samples Tested			
Fresh Strawberry	4/4	4/4	0/4	0/4
Frozen Strawberry	4/4	4/4	0/4	1/4
Fresh Raspberry	3/4	2/4	2/4	1/4
Frozen Raspberry	1/4	1/4	0/4	0/4

Samples are considered positive if the Cq is less than or equal to 40. For each concentration and matrix, four samples were treated with the ApoH methods. No statistical analysis was performed.

**Table 3:** Detection of NoV GII.4 on berries using capture by ApoH beads.

Food	Genome Copies/Sample			
	10^5^	10^4^	10^3^	10^2^
	Number of Positive Samples/Number of Samples Tested			
Fresh Strawberry	4/4	0/4	1/4	0/4
Frozen Strawberry	4/4	1/4	2/4	0/4
Fresh Raspberry	2/4	2/4	0/4	1/4
Frozen Raspberry	1/4	0/4	2/4	0/4

Samples are considered positive if the Cq is less than or equal to 40. For each concentration and matrix, four samples were treated with the ApoH methods. No statistical analysis was performed.

## Data Availability

The sequence of the norovirus GII.4 used in this study is available via GenBanK as number MZ357344. The raw data supporting the conclusions of this article will be made available by the authors without undue reservation.
